# Life with an Indwelling Urinary Catheter: Experiences from Male Patients Attending the Urology Clinic at a Tertiary Hospital in Northwestern Tanzania—A Qualitative Study

**DOI:** 10.3390/nursrep12040077

**Published:** 2022-10-26

**Authors:** Asteria L. M. Ndomba, Rose M. Laisser, Eveline T. Konje, Joseph R. Mwanga, Stephen E. Mshana

**Affiliations:** 1 Archbishop Anthony Mayala School of Nursing, Catholic University of Health and Allied Sciences, Bugando Area, Mwanza P.O. Box 1464, Tanzania; 2 Department of Biostatistics, Epidemiology and Behavioral Sciences, Catholic University of Health and Allied Sciences, Bugando Area, Mwanza P.O. Box 1464, Tanzania; 3 School of Public Health, Catholic University of Health and Allied Sciences, Bugando Area, Mwanza P.O. Box 1464, Tanzania; 4 Department of Microbiology and Immunology, Catholic University of Health and Allied Sciences, Bugando Area, Mwanza P.O. Box 1464, Tanzania

**Keywords:** lived experiences, catheter users, information, outpatients, long-term indwelling urinary catheter, Tanzania

## Abstract

Experiences from patients living with a long-term indwelling urinary catheter (IUC) at home among men attending urology clinics have not been reported. Evidence-based information on such experiences is important for improving nursing care in low- and middle-income countries such as Tanzania. Using a descriptive phenomenological qualitative research design, we observed two main themes: “*Adjustments to positive living with a catheter at home*”, denoting that social interaction and psychological and spiritual support are important to positively live with the catheter, and “*The home environment influences negative or positive living*”, considering intimacy and altered body image to significantly influence the ability to practice sex, leading to wives’ self-sacrifice. Respondents experienced difficulties in living with a long-term IUC at home due to a lack of information from healthcare professionals on how to manage their catheters and urine bags. In adjusting to positive or negative living with a catheter at home, respondents had to figure out strategies to minimize psychological and emotional trauma and hasten the adjustment process. A clear guideline or checklist is needed to ensure that all important information is provided by health professionals at the time of discharge and during subsequent visits to patients on how to care for the catheter insertion sites and help them adjust to living with a long-term IUC.

## 1. Background

An indwelling urinary catheter (IUC), urethral or suprapubic, is commonly used to manage patients with incontinence and urine retention due to benign prostatic hypertrophy (BPH) and other neurological diseases. According to Atakro et al. [1], though intermittent catheterization is the gold standard for the management of urinary problems in men, many men in Africa, including in Tanzania, are managed with indwelling urethral catheters. The majority of clients in Africa with BPH tend to live with a long-term IUC due to the inability to pay for prostatectomy [2,3]. Common problems experienced by those living with a long-term IUC include urinary tract infections (UTI), kidney stone formation, painful bladder spasms, blockages, and leakages [4,5,6,7]. However, there are other non-infectious complications such as loss of dignity, loss of job, erectile problems, diminished desire for sexual intercourse, and loss of money through hospital bills [1]. All these have a serious impact on the social and psychological well-being of patients living with a long-term IUC at home.

One study of long-term catheter users older than 65 years revealed that older people adapt to living with a catheter as a consequence of either being “at ease” or “uneasy” with the catheter, while other studies report that catheter users appear to adjust following a period of transition, taking possibly up to a year to adjust to it [8,9]. A number of studies have reported that many people living with an IUC at home [8,9,10,11] have not been well prepared to live with it. An IUC, according to Adomi et al., is defined as urinary catheterization for 28 consecutive days or longer. However, a long-term IUC, according to our study was defined as having an indwelling Foley’s urinary catheter >14 days in situ continuously. Researchers writing about patients’ experiences of living with a long-term IUC have often noted that catheter users wanted and would benefit from more information on living with a long-term IUC [8,9].

Life with a catheter at home is a transition and needs support due to the uncertainty and possible stress associated with it. Brandburg documented that nurses occupy a primary role in assisting older adults to make a healthy transition and to successfully adapt to the home environment. Nurse–patient relationships often are built during times of transition involving developmental, situational, or health-status changes [12]. Sexuality and body image considerations are important in the day-to-day life of a patient with a long-term IUC, though these issues are hardly discussed due to taboos, beliefs, and fear of offending the patients. As narrated by Barker-Green, urinary catheter users are particularly in need of nursing support in acknowledging their sexuality due to the physical placement of the medical device; the insertion of a urinary catheter affects self-esteem and body image and patients often feel ill-prepared to deal with this change [10].

According to the study done by Ndomba et al., in Tanzania, the prevalence of patients living with an IUC at home was 9.6% [2]. This prevalence might increase in Tanzania since the number of older people aged 60 years and above who are subject to using an IUC might increase to over 7.7% in 2050 [2]. Assessing patients’ sexuality is part of any holistic assessment, especially for people living with a long-term IUC. According to Saunamaki and Englestrom, nurses declined to talk to patients about sexuality due to their own emotions, discomfort, and the belief that the subject is taboo [11] and that people tend to think that older people are not sexually active. However, as reported by Laumann et al., most older people are sexually active [13].

Knowledge of the prevalence of patients living with a long-term IUC is now established in Tanzania, yet their lived experiences, their needs, and their concerns about living with it in their day-to-day life are not known in Northwestern Tanzania. Understanding their life experiences with an IUC at home is crucial in addressing their needs and concerns with healthcare professionals. As evidenced by a study done by Alex et al., only 15 studies from the USA, Australia, UK, and Turkey were published between 2003 to 2019 that focused on the needs of patients living with an IUC and their quality of life [14]. There are no studies from Sub-Saharan Africa, including Tanzania, which have explored the experiences of patients living with a long-term IUC at home. Therefore, this study aimed at exploring the lived experiences of participants with a long-term IUC living at home in Northwestern Tanzania. To the best of our knowledge, this is the first study to be conducted in Sub-Saharan Africa, particularly in Northwestern Tanzania, that has shed light on the experiences of participants living with a long-term IUC at home.

## 2. Methods

### 2.1. Study Design and Setting

#### 2.1.1. Study Design

The study was conducted between July 2017 and September 2017 with eleven (11) outpatients living with a long-term IUC. This was a descriptive phenomenological study that sought to investigate the everyday lived experiences of participants living with a long-term IUC at home in Northwestern Tanzania. Edmund Husserl’s philosophy was followed, where the researchers collected and analyzed the data while suspending their preconceived assumptions about the phenomenon (Bracketing) to gain deeper insights into how participants viewed and understood those experiences [15].

#### 2.1.2. Study Setting

The study was done at the urology clinic at Bugando Medical Center (BMC), which is a consultant, tertiary, and teaching hospital located in the Northwestern Lake Zone of Tanzania. BMC has a bed capacity of 1000 with nine (9) outpatient clinics, including the urology clinic. This hospital serves eight (8) regions, namely the Mwanza, Simiyu, Mara, Kagera, Shinyanga, Geita, Tabora, and Kigoma regions, with an estimated population of 13 million people, among whom 27.7% are 60 years and above.

### 2.2. Participants and Eligibility Criteria

Eleven (11) long-term indwelling urinary catheter users were recruited using a purposive sampling technique, as described previously [15], from the 202 participants living with a long-term indwelling urinary catheter at home who were attending the urology clinic at Bugando Medical Centre (BMC). The profile of the participants reflected the heterogeneous population of long-term catheter users, which included eleven men ranging in age from 22 to 85 years. The participants included both urethral and suprapubic catheter users. The sample had varying durations of catheter usage and a range of self-reported reasons for long-term catheterization. Inclusion criteria were adults using an indwelling catheter (suprapubic or urethral) for more than 14 days continuously.

The selection of the participants was based on who could best inform the research questions and enhance the understanding of the phenomenon under study. The participants were chosen based on their experiences with the phenomenon under study but varied in characteristics and in their individual experiences to increase the possibility of shedding light on the phenomena under study from a variety of aspects. The participants lived in urban, suburban, and rural settings.

### 2.3. Data Collection

The first author (ALMN) spent time in the urology clinic as well as in the patients’ homes to be well-oriented with the informants’ natural settings. She conducted all the interviews using an in-depth interview guide with flexibility. The in-depth interview guide was prepared in the Kiswahili language (the author’s national language) in an effort to accurately capture the experiences of long-term catheter users. Six in-depth interviews were conducted in the urology clinic, whereby a private room was designated in collaboration with the nurse in charge of the urology clinic. The remaining five (5) in-depth interviews were conducted in the participants’ homes after agreement between the researcher and the interviewees on a convenient time and date. Information from participants’ experiences was collected, allowing each participant to highlight his/her own concerns, meanings, and priorities. The interviewer had good communication skills to hear what was being said so that participants were able to recount their experiences as fully as possible without unnecessary interruptions. Adopting open and emotionally neutral body language encouraged participants to express themselves.

Supplementary questions were prompted depending on particular areas, including any important issues identified through the literature review. At the end of the interview, participants were thanked for their time and were asked if there was anything they would have liked to add. This gave respondents an opportunity to deal with issues that they had thought were important but had not been dealt with by the interviewer. This led to the discovery of new, unanticipated information.

These interviews were conducted face-to-face in privacy (i.e., with the participant and the principal investigator for confidentiality purposes) each taking 30 to 45 min, and were conducted only once. All patient identifiers were removed from transcripts. Demographic information was also collected from each participant.

### 2.4. Research Tool

We prepared an interview guide with eleven themes coupled with relevant questions related to the central theme (Supplementary file S1). These included reasons that led participants to have an indwelling urinary catheter, experiences of how to live with a catheter, and how participants managed their indwelling urinary catheters and the urine bag, including everyday patterns of living with a catheter. The tool also included asking participants about the information that was given regarding living with an indwelling urinary catheter at home and their relatives’ perceptions towards their life with a catheter. The development of the guide was based on the general understanding of indwelling urinary catheters. During data collection and initial analysis, this pre-understanding was put within brackets [15]. Open-ended questions were used to elicit patients’ views and experiences of living with an indwelling urinary catheter (IUC). These questions were the same for all the participants enrolled in the study.

Written consent was obtained from each participant prior to participation in this study. Each interview began with a brief overview of the purpose of the study, followed by an open-ended question, for example, “May we discuss about how it is for you to live with a catheter?” In order to have a full documentation of the interview data, we recorded all the interviews using digital recorders. The recording of the interview made it easier for the researcher to focus on the interview content and the non-verbal cues that aided in data interpretation.

### 2.5. Analytic Procedures

The in-depth interviews were transcribed verbatim in Swahili to enable preliminary analysis. Later, we translated them into English to facilitate peer-debriefing sessions and joint analysis in the research group. During the translations, we took care to accurately convey the informants’ words and intentions and to explain metaphoric expressions. All four researchers were conversant in both English and Swahili and were able to confirm the translated meanings.

In keeping with the interpretive description approach, data collection and analysis was an iterative process. Recorded interviews (audio files) were transferred into a computer and transcribed verbatim by two independent transcribers. For the sake of quality control, the back-translation of the transcripts was performed. Since we had a small data set (i.e., 11 interview transcripts), data analysis was carried out manually by two investigators independently for the sake of quality control, as follows: During preliminary analysis, research topics from an interview guide were used for initial categorization. Categories were inductively developed from the data. The topics were then defined as codes (labels) and used to further code the data, and the codes were developed manually by two investigators. Data were arranged into broad categories (i.e., coded categories) known as codes. Data were systematically and iteratively reviewed to ensure an exhaustive set of data to support each code. This process allowed for the identification of potential additional themes. An iterative thematic content analysis was performed on the data. This involved finding patterns of meaning by identifying codes or concepts, which were then built into themes. Two major themes emerged from the data, namely: adjustments to positive living with a catheter at home and the home environment’s influence on negative or positive living.

Quotes were taken where necessary to support the findings. Two investigators interpreted the data to allow for transparency and avoid one’s position influencing interpretation. The two investigators assigned more or less the same meanings to the data. All data were manually analyzed by the research team.

### 2.6. Ethical Considerations

The study was conducted according to the guidelines of the Declaration of Helsinki and approved by the Research Ethics Committee from the Joint Catholic University of Health and Allied Health Sciences (CUHAS)/Bugando Medical Centre (BMC) Research Ethics and Review Committee (CREC) with ethical clearance number CREC/152/2016. Study aims and objectives, procedures, and potential risks and benefits (full disclosure) were explained to participants through the information sheet read/provided to them. Adequate written informed consent was obtained from all subjects involved in the study through signatures or thumbprints on the consent forms. Anonymity, confidentiality, and privacy were highly maintained, and participants were informed that participation in the study was on a voluntary basis and reminded of their rights to refuse to take part in the study or to withdraw from the study at any time during the study without any consequences. In this study, consent was not regarded as “one-off event” but rather a process that was negotiated and renegotiated throughout the entire research process.

### 2.7. Trustworthiness

All four research team members are Tanzanians and fluently speak the local language of Kiswahili. To increase credibility, the research team made repeated visits to the study site. Prolonged engagement with the study site by ALMN helped to build trust in the community surrounding these participants with a long-term IUC. Preliminary findings were subjected to checks with two rural residents in the Misungwi-Mwanza region to confirm the meaning of certain local expressions. The research team also had continuous peer debriefing as the study progressed. A flexible guide, phenomenological design, multidisciplinary research team, verbatim transcription, and predefined analytic procedures were used to promote study rigor. During the analysis, the fitness and relevance of the emerging categories to the research question were tested by constant comparison and checking between the text, codes, and categories and by paying specific attention to outliers or negative cases.

## 3. Results

### 3.1. Socio-Demographic Characteristics of the Study Participants

Eleven (11) participants were interviewed. Their ages ranged from 22–85 years. All were males. Many of the participants were married and their main occupation was subsistence farming. The majority of participants had attained a secondary level of education. Three males had a suprapubic catheter and eight had a urethral catheter. Participants needed a catheter for various reasons; ten had an inability to pass urine due to enlarged benign prostate hypertrophy (Table 1).

### 3.2. Experiences of Patients Living with a Long-Term Indwelling Urinary Catheter at Home

During analysis, two main themes emerged: 1. “*Adjustments to positive living with a catheter at home*” and 2. *The home environment influences negative or positive living*. Living with a catheter was somehow difficult, however, respondents reported how positively they managed to continue with it.

The first theme, “*Adjustments to positive living with a catheter at home*”, occurred with four sub-themes, namely: information on catheter management at home, social interaction, psychosocial and spiritual support, and sanitation and infection prevention. The second theme, “*The home environment influences negative or positive living*”, occurred with three sub-themes, namely: intimacy and body image, bowel elimination difficulties, and psychological and emotional distress resulting from the surroundings. Below are the detailed narrations of experiences and quotes to exemplify.

#### 3.2.1. Adjustments to Positive Living with a Catheter at Home

Respondents categorized their adjustments during the day and night.

##### Day-Time Adjustment

All respondents wore clothes according to their chosen style, such as trousers with wider legs and pockets, shorts (bukta), kanzu, or kitenge, to help disguise the drainage bag’s presence due to shame, humiliation, and loss of dignity. The following quotes from participants say it all:

*“I wear kitenge with shorts (bukta) inside… It has to be kitenge… even when I go to the hospital I put on kitenge.”*

(PIUC 4)

*“I am hiding the urine bag and the tubing in my trousers’ pocket to prevent people and even children saying eeh what is wrong with this old man? I would feel very embarrassed. But now people do not know that I have a catheter with me.”*

(PIUC 6)

##### Night-Time Adjustment

All respondents positioned their urine bags either on the floor wrapped in a cotton material or in a bucket to prevent accidents such as bursting and leakage during the night to avoid sleep disturbance. Two different respondents had different remarks, as follows:

*“I keep it aside like this on the floor… I sleep on the floor with a mattress on… So when I wake up I check if it is full or not and if it’s full I wake my relative to escort me. I walk slowly to the toilet where I empty the urine bag. I don’t sleep well because I feel pain.”*

(PUIC8)

*“I was worried if I keep it on the bed and it becomes full, I may sleep over it making it burst… I put the urine bag in the bucket beside my bed then sleep.”*

(PIUC 12)

##### Information on Catheter Management at Home

Adjustment to living with a catheter in a positive way was thought to be important. The respondents reported a lack of such information from the time they were discharged home. One participant lamented:

*“No instructions were given to me… I had to figure out myself on how to handle the urine bag when going to sleep. I am placing the urine bag in the bucket; so, when it bursts the urine goes in the bucket. I do not know how frequent I am supposed to change the urine bag nor the catheter.”*

(PIUC 6)

##### Social Interactions

Interaction with others is important in mediating the relationship a person has with others in the family. Respondents felt isolated because they could not go to places or visit their friends due to their body image while living with an indwelling urinary catheter. However, respondents expressed gratitude for their friends and neighbors who came to visit them and brought them necessities for daily living for those who were too old. The following remarks from two participants drive the point home:

*“Having a catheter is the most distressing aspect of my daily life”—“One thing I am not happy about is to move along with this bag, sometimes I feel that people look at me as unclean.”*

(PIUC 2)

*“I cannot mingle with people like watching football matches with my catheter on, or even to visit friends… for now I am just at home.”*

(PIUC 5)

##### Psychosocial and Spiritual Support

Physical and psychological support, particularly from spouses, was described as important by all respondents. All respondents acknowledged the support they received from their spouses and from religious groups for their moral support in living with a long-term IUC, as these minimized the spiritual distress from not attending prayers in the church or mosque. On religious support, one participant remarked:

*“I am a Muslim and I pray 5 times a day and I have to go to the mosque… Friends and Sheik come to pray with me at my house… they helped me psychologically to bear with my problem.”*

(PIUC 4)

Regarding family support, one participant said:

*“I am getting support from my wife. She is the one who is giving me a lot of support. She is encouraging and comforting me that the problem will end.”*

(PIUC 9)

##### Sanitation and Infection Prevention

The prevention of infection through cleanliness and good sanitation was also noted to be useful when living with an indwelling catheter. However, respondents expressed dissatisfaction with a lack of guidance given by health professionals on how to maintain the cleanliness of the catheter insertion sites, leading to feelings of humiliation and depression on the part of suprapubic catheter users. Few participants reported having good modern toilets with good water supply, necessitating good hand hygiene in preventing catheter-associated infection. The majority reported having pit latrines and some went to the bush with no cleaning facilities such as water and soap. With regard to cleanliness, one participant said:

*“Nurses told me cleanliness is very important on my wound (suprapubic site), but they did not tell me how to clean myself when I stay with a catheter… I had perineal wounds the situation was not good. The bandage which was kept on the insertion site—wound, was stained with blood and water I was using while taking a bath… I stayed with it for a week.”*

(PIUC 4)

#### 3.2.2. The Home Environment’s Influence on Negative or Positive Living

##### Intimacy and Body Image

Sexual relationships are described as a necessity for human beings. However, the majority of respondents reported that their sexual drive was down and they had no desire for sexual intercourse as the catheter was a hindrance; they reported having understanding wives. Few reported to have felt the need to have it but could not do it. One participant remarked:

*“I have the desire for sex but how can I do it comfortably with this catheter?”*

(PIUC 12)

In the same vein, another participant said:

*“I get sexual desire… sexual drive is not that strong… you know I am a priest so I do not dwell on it; I just let it go.”*

(PIUC 6)

##### Bowel Elimination Difficulties

Normal bowel movements are described as important for physiological well-being. The majority of the respondents reported having normal bowel movements in pit latrines or in the bush with inadequate hand-washing facilities (water and soap), and few reported having modern toilets with an adequate supply of water and soap for hand-washing. However, they reported having difficulties in assuming the squatting position with a catheter on when using eastern-type toilets and pit latrines. Some reported having an insufficient intake of fruits, vegetables, and water, leading to constipation and pain. On bowel movements, the following four participants remarked:

*“The environment is safe, clean, with plenty of water. The toilet I am using is western type. Had it been the eastern type, I would not have been able to use it. Squatting is not easy with a catheter on. Facilities like soap, sanitizers is not a problem. Washing of my hands after attending to the call of nature is not a problem to me.”*

(PIUC 6)

*“When I go for a long call, I don’t go to the toilet, I just go to the bush, because it is not close to home. To the bush I go with water in a small can for cleaning myself… not with soap.”*

(PIUC 5)

*“I defecate in forest and I use tree leaves to wipe myself. The whole family goes to the forest.”*

(PICU2)

*“I feel pain… defecation is a problem when I am in a squatting position… some other times it fails to come out, small liquid comes out… that is one of the disadvantages, but the urine is coming out that is the advantage. The food we use ugali (stiff porridge), rice…. Vegetables I just eat normally… to be honest… fruits… no.”*

(PIUC 8)

##### Psychological and Emotional Distress Resulting from Surroundings

Minimizing the psychological and emotional trauma resulting from living with a long-term indwelling urinary catheter at home by health professionals is important.

The majority of respondents expressed dissatisfaction with living with a long-term indwelling urinary catheter due to inadequate help from healthcare professionals in minimizing psychological and emotional distress leading to despair, frustration, job insecurity, and feelings of imprisonment. The frustrations of living with a catheter were real, as evidenced in the following remarks of participants:

*“If I get cured early and this catheter is removed… I will be very happy. I am like a prisoner with this catheter. If I had no catheter, I would be doing my activities to earn my living.”*

(PIUC 5)

*“I am a retired officer I cannot be called back to work even if they wanted me too… I cannot because of this catheter…. It is embarrassing.”*

(PIUC 12)

*“Currently I cannot even go to work because at work we have to put on a uniform. I do not have neither money nor health insurance to quicken my treatment. I’m wondering for how long I am going to be in this situation. They keep on postponing and giving me further dates because I do not have money to pay for operation to remove the enlarged prostate gland. I cannot do anything because I am sick.”*

(PIUC 7)

## 4. Discussion

This phenomenological. descriptive study emanated from our three previous studies [2,16,17] in relation to long-term indwelling catheters to gain an understanding of the quality of life of these patients. The study highlights the lived experiences and the perceptions of the participants living with a long-term indwelling urinary catheter (IUC) and how positively or negatively they were able to make adjustments to their life in overcoming the daily encountered challenges. As regards adjustments to positive living with a catheter at home during day time, all our respondents felt the need to incorporate the catheter into their lives by employing different strategies of concealing their catheter and urine bags by wearing trousers with wider legs and pockets, shorts (bukta), kanzu, or “kitenge” to help disguise the drainage bag’s presence. The presence of the drainage bags gave the participants feelings of low self–esteem due to shame, humiliation, and loss of dignity. These findings are similar to the study done by H. Godfrey in the UK, whereby participants preserved their self-esteem by employing strategies such as safeguarding their dignity by concealing the catheter by wearing long skirts to avoid embarrassment [18]. A similar finding was noted in a study done by Fowler et al. in Bristol, UK. Negative comments were related to the catheter bag, and participants described how they tried to conceal its presence [8].

Regarding nighttime adjustments, all respondents reported employing a variety of strategies to manage daily life, including not drinking much when going to bed with the intended result of not needing to wake up frequently to empty the urine bag. The respondents also talk about positioning their urine bags either on the floor wrapped in a cotton material or in a bucket for fear of wetting themselves in case of accidents such as the bursting or leakage of the urine bag during the night, thus ending experiences of sleep disturbance which were echoed by the respondents. According to Fowlers et al. [8], the theme of nighttime management has not previously been identified. Participants independently adopted similar strategies when adjusting to sleeping with a catheter. The frequent use of a bath towel to protect the bed was reported in addition to the experience of damage to bedding and the bed from urinary leakage. Similarly, the same was observed in a study done by Fowlers et al. [8] whereby participants talked about interrupted sleep due to concerns or problems with catheter drainage bags. In contrast, others reported an improved sleep pattern, as they no longer had to get up at night to urinate. Most placed the overnight drainage bag in a bucket rather than use a stand because of a past experience with a leaking or ruptured bags. It is high time for nurses and doctors to consider these non-infectious complications, which have been given the least consideration in the management of patients with a long-term IUC, as they seriously affect the well-being of these respondents in their overall quality of life (QoL). Removing or treating the underlying problem that leads to prolonged living with an indwelling urinary catheter as soon as possible would be a solution to all these non-infectious complications.

This study has revealed that social interaction with others is very important in mediating the relationship a person has with others in the family and those around them and in their neighborhood. Living with a long-term indwelling urinary catheter without knowing when they could be freed from it, respondents feel isolated and regard themselves as prisoners because they cannot go to places or visit their friends due to their change in body image. Our findings are similar to the study done by Kralik and colleagues, whereby respondents felt socially and psychologically incapacitated and further documented that having a catheter also initially affected physical closeness with extended family such as grandchildren [19]. However, in our study, our respondents expressed gratitude for their friends and neighbors who come to visit them and bring them necessities for daily living for those who are too old.

This study has revealed differences in participants’ personal experiences of adjusting to living with long-term catheterization. This study highlights the importance of practical and psychological support from family, particularly from spouses. Physical and psychological support, particularly from spouses, was described as important by all respondents. All respondents in this study acknowledged the support they received from their spouses and from religious groups for their moral support in living with a long-term IUC, as this supports minimized the spiritual distress from not attending prayers in a church or mosque. Those without spouses also acknowledged the importance of the support they received from friends and religious groups.

Another important area revealed in this study was a lack of information from healthcare professionals about managing the IUC at home. Adjustment to living with a catheter in a positive way was thought to be important. All respondents in this study reported a lack of information from the time they were discharged, and as a result, they had to figure out how to cope with the challenges they faced, such as how to handle the urine bag when going to sleep *or* how to maintain clean insertion sites, especially the suprapubic site, in order to prevent infection. Participants are keen to understand more and they feel that they were not given enough information to act upon. According to Godfrey H, the support necessary to help patients self-manage their conditions goes beyond giving information and involves healthcare professionals helping patients build confidence and make choices that result in improved self-management [18]. These findings are also similar to what Prinjah et al. reported [9]: “67% of partcipants stated that no one had initially explained to them how to care for their catheter; 42% could not say how their catheter worked”. Moreover, Murphy et al. documented that “patients often report lack of knowledge and support with managing and making day-to-day decisions about their catheter” [7]. This lack of information in our study might have led the respondents to have adverse outcomes such as infection at the insertion sites, especially the suprapubic site, as evidenced by narration about the presence of pus draining from these sites and not knowing how to clean them. The issue of infection in these respondents with a long-term IUC has also been documented in a recent study by Ndomba et al. [20,21], whereby “the prevalence for CAUTI was 82.2% for outpatients living with a long-term IUC at home”. With this high prevalence of CAUTI and considering the low socio-economic status of the participants, as observed also in the study done by Ndomba et al. [20], where “the quality of life was poor in all the domains especially the psychological domain”, it is not surprising that living with an indwelling urinary catheter causes strain on the individual, physically, socially, psychologically, and environmentally. As a result, these respondents express that they are not happy; they are uncomfortable and have lost hope in their lives.

Other experiences that this study has illuminated include psychological and emotional distress due to inadequate information and help from healthcare professionals in prompting early treatment for the underlying problems necessitating the prolonged use of an IUC. Participants in this study feel that the stress, humiliation, shame, job insecurity, neglect, abuse, resentment, and postponement of treatment are contributed to by healthcare systems that do not have a mechanism for minimizing the prolonged stay with an indwelling urinary catheter, leading to all this emotional and psychological distress, which are reported to affect their overall well-being and their quality of life. Participants express how to minimize the psychological and emotional trauma from living with a long-term IUC at home. These findings are similar to a study that was done in Ghana, where they reported that “the use of the indwelling urethral catheters led to reduced activities and loss of jobs” [1]. The outcomes of these findings in our study could also have an impact on the quality of their life, as evidenced by a recent study by Ndomba et al. [20], where they found that “the quality of life of the patients living with a long-term IUC at home in Tanzania was poor in all domains especially the physical, the psychological and environmental domains”. The inadequacy of the information given to participants by health professionals to enable them to experience and adapt to a positive life with an IUC at home could possibly be associated with a lack of awareness of the kind of life these participants undergo at home in living with a long-term IUC. Therefore, there is a necessity for doctors and nurses to be more aware of the needs of patients living with a long-term IUC. Safdar et al. [5] documented that “health care workers involved in catheter care should undergo training on how to properly engage and educate patients because lack of knowledge about indwelling urinary catheters is likely to be a major contributing factor in patient decision-making regarding urinary catheters”.

Socialization in this study was affected due to the presence of the catheter they were wearing, which affected their body image, as participants reported feelings of isolation, that they could not go to places or visit their friends, and feeling like prisoners. These findings are also evidenced in a study done in Ghana where all respondents in that study stated that “the IUC reduced their socialization with friends and family because of the embarrassment the catheter caused when it leaked. They smelled of urine even when they were well dressed” [1]. Likewise, Fowler et al. [8] documented that “people who reported difficulty adjusting to a long-term IUC frequently described themselves as socially isolated and were more likely to have poor support systems and multiple conditions”. Catharina et al. also documented, on loneliness and restrictions in daily living, that “restrictions in life influence the mood: “you become grumpy” and depressed and even irritated” which leads to the avoidance of social interaction [22].

Psychosocial and spiritual support from their spouses and religious groups are paramount while living with a long-term IUC, as reported by all respondents. This has been deemed important to them in minimizing spiritual distress and gaining moral support. This finding correlates to a recent study on quality of life by Ndomba et al. [20], that “quality of life (QoL) in the social domain was slightly better compared to other domains”, and this was due to the support from their spouses/significant others. Regarding spiritual support, as echoed by one of the respondents, ”it has helped me psychologically to bear with my problem”; this finding has also been observed in a study by Grossoehme et al. and Harris et al. [16,23]: “spiritual care is a fundamental component of all high-quality compassionate health care, and it is most effective when it is recognized and reflected in the attitudes and actions of both patients and health care providers. Supporting the everyday spiritual rituals of the patient such as reading, meditating or praying are important to the spiritual well-being of patients and should be supported”.

Few respondents reported experiencing bowel elimination difficulties, specifically constipation, which led them to experience pain, as well. These findings are similar to a study by Wilde et al. [17], whereby only a few persons were constipated. Constipation in our study respondents could be attributed to the type of food they were eating, inadequate fluid intake, and a lack of fruits in their diet. They narrated eating “*ugali*” (stiff porridge), most probably polished maize flour lacking husk (fiber), which is important for fecal elimination. The pain they were experiencing could be due to bladder spasms resulting from constipation.

Sexual relationships are described as a necessity for human beings, including those older participants living with a long-term IUC, despite the difficulties in meeting this need, as the catheter is a hindrance. However, the majority of respondents reported that their sexual drive was down and they had no desire for sexual intercourse, as the catheter was a hindrance; they reported having understanding wives. Few reported having felt the need to have it but could not do it. These findings are similar to the study by Sweeney et al. [24], who documented that “some participants reported difficulties with sexual expression and impaired perception of sexual self since the insertion of their suprapubic catheters. The literature proposes that sexual intercourse may be easier for those who have a suprapubic catheter compared to a urethral catheter”.

## 5. Study Limitations

The study results cannot be generalized to all areas of Tanzania (or Africa); however, they are in line with other studies from other parts of the world, though few. In addition, there were no women participants living with a long-term IUC enrolled in the study, although the literature documents that long-term indwelling urinary catheterization is usually indicated for older men and women, hence further study on lived experiences for women is recommended. Furthermore, almost all the participants were older men, therefore, it is possible that they were not very open when discussing sexual relationships due to cultural reasons as well as taboos.

## 6. Conclusions

To the best of our knowledge, this is the first study in northwestern Tanzania that has shed light on the experiences of patients living with a long-term IUC at home. Respondents reported experiencing difficulties in living with a long-term IUC at home due to a lack of information from healthcare professionals on how to manage their catheters and urine bags. In adjusting to positive or negative living with a catheter at home, respondents reported using various strategies in their lived experiences of adjusting to positive or negative living with a long-term IUC at home during the day—by concealing their catheter and urine bags by wearing trousers with wider legs and pockets, shorts (bukta), kanzu, or “kitenge” to help disguise the drainage bag’s presence due to shame, humiliation, and loss of dignity—and during the night—by positioning the urine bags in buckets or directly on the floor, either naked or wrapped in a piece of cotton material for the easy absorption of urine in case of leakage. They acknowledged the support they received from their families and friends, which minimized the psychological and emotional trauma from long-term catheterization and also hastened the process of adjustment through their love and support (morally and materially).

We recommend that healthcare professionals have clear guidelines or checklists to ensure that all important information during discharge home and during subsequent visits is communicated to patients on how to care for the catheter insertion sites and help them adjust to living with a long-term IUC.

A multicenter study is warranted to explore the experiences of patients living with a long-term indwelling urinary catheter at home in Tanzania and other sub-Saharan African countries for improving nursing care for their well-being.

## Figures and Tables

**Table 1:** Participants’ socio-demographic characteristics.

Characteristics	Number
**Religion:/Christian**	8
**Religion/Moslem**	3
**Marital status/single**	1
**Marital status/married**	10
**Marital status/divorced**	1
**Job/subsistence farmers**	11
**Level of education/primary**	11
**Cather type/urethral**	8
**Catheter type/suprapubic**	3
**Cather use in months**	2–24
**Indication/BPH**	10
**Injury to pelvic region**	1

## Data Availability

The data are available upon request and the request should be made to the Director of Research and Innovation at the Catholic University of Health and Allied Sciences.

## Supplementary Materials

The following supporting information can be downloaded at: https://www.mdpi.com/article/10.3390/nursrep12040077/s1, Supplementary file S1: Interview guide.
