# Supplementary material for: Life with an Indwelling Urinary Catheter: Experiences from Male Patients Attending the Urology Clinic at a Tertiary Hospital in Northwestern Tanzania—A Qualitative Study
Source: Nurs Rep. 2022 Oct 26;12(4):791–803. doi: 10.3390/nursrep12040077 (PMC9680475; doi:10.3390/nursrep12040077)
Supplement: Supplementary file 1 [file nursrep-12-00077-s001.zip › nursrep-1934122-supplementary.pdf]

Supplementary file S1: Interview guide

Introduction – researcher introduces herself and the study. This is followed by the following questions if the respondent is ready.

1. **May I ask you information about yourself? (Demographic Information)**
    - Age
    - Sex
    - Marital status
    - Position in the family
    - Occupation: Peasant/employed(position at work if any)/self-employed
    - Religion
    - Residence: urban/rural
2. **What led you to have an indwelling urinary catheter you are using?**
    - **May we discuss** What were the reasons for you having the indwelling urinary catheter?
    - How did you feel when you first heard you would need a catheter?
    - What was important to you then?
    - What is important to you now?

3. **May we discuss about the catheter you are using**
    - What type of indwelling catheter do you use?
    - Have you used different types?
    - How long have you been using a urinary catheter?
    - How have you felt about the different types of catheters you've used

4. **May we discuss about how it is for you to live with a catheter.**

    - What is it like to use a urinary catheter?
    - How does the catheter affect your everyday life?
    - How has the catheter influenced your leisure/work/social life/finances/relationships?
    - How has using a catheter affected your friends and family
    - What difficulties do you experience living with a catheter?
    - What kinds of problems do you have associated with the catheter?
    - Who do you talk to about your experience of the catheter?

5. **Can we talk about how you manage your indwelling urinary catheter and the urine bag?**

    - What steps do you/family take to manage your catheter and urine bag?
    - What do you think are the most important aspects of keeping your catheter working well?
    - Who helps you to empty the urine bag?
    - Who helps you with your catheter and the urine bag?
    - How often do you have help with your catheter and urine bag?
    - How do you position your urine bag while sitting, walking, visiting friends, going to church/mosque and sleeping?

6. **Can we talk about your everyday pattern of living with a catheter?**

    - Benefits of using it compared to not having it
    - How does the catheter affect your everyday living?

    - What changes have you had to make in your day-to-day life with the catheter?
    - Changes in his/her activities of daily living when using the catheter
    - Changes in caring others
    - Changes in her/his income

- Changes/differences in relationship with husband/wife or other significant others
- Differences in the job?
- Comments on social life, spiritual life?

7. **Can we talk about catheter issues pertaining to your experiences about living with an indwelling urinary catheter at home?**
   - How do you know when your urine bag is full?
   - How do you manage with the urine bag when you're away from home?
   - Where do you buy the drainage urine bags in case you need them?
   - What do you think are the most important aspects of keeping your catheter working well?
   - How do you manage problems with blockage, leaking, discomfort, pain, bursting and sleep?
   - How often is the catheter changed?
   - How do you feel before/after a change?
   - How have you learned to manage your catheter?
   - What sort of a catheter type would you like to have?
   - How could the current design be improved

8. **Can we talk about the information given to you about living with an indwelling urinary catheter at home?**
   - How have you felt about the information you were given by doctors and nurses at different stages (at discharge and during scheduled visits) on how to care for your catheter at home?
   - What would you liked to have known at various stages?
   - What information would you like now but don't have?
   - Where do you get (formal and informal) support?
   - How have you felt about the (formal and informal) care and support you've had?
   - How have you felt about the support you get from your family, relatives and friends?
   - What information were you given about living with an indwelling urinary catheter at home?
   - ➢ How to keep the urine bag while sitting , walking or sleeping
   - ➢ How to maintain cleanliness of the catheter and the insertion site.
   - ➢ How to empty the urine bag
   - ➢ What to eat and drink

9. **Can we talk about your responsibilities regarding maintaining/keeping the indwelling urinary catheter?**
   - How do family members support you in maintaining/keeping the indwelling urinary catherter?

   - Responsibilities to own care of the indwelling urinary catheter
   - When it is supposed to be changed
   - Impact on lifestyle in relation to engaging others on support
   - Other comments to strengthen support to living with indwelling catheter

10. **What can you say about safety and prevention of infection when using the catheter?**
    - How is your environmental sanitation of the house?
    - What type of toilet do you have?
    - How is the availability of water and soap in your home?
    - How is hand hygiene practiced now that you have the indwelling urinary catheter?
    - What type of housing do you have in the context of hygiene?
    - What is your opinion regarding safety preventive measures of infection?
    - What is your opinion on future life on living with a catheter?
    - What is the role of other family members' support and care

11. **Can we talk about how you feel telling your family and friends that now you are using an indwelling urinary catheter for emptying your bladder**
    - How have family and friends reacted?
    - Have you had a lot of support?
    - Are there any people you don't like to tell?
    - Is there anything else that you would like to tell me?

12. **Can we talk about conveying anything that you think is important for other catheter users/to carers/to health professionals to be aware of?**
    - Are there any messages that you'd like to give to other catheter users/to carers/to health professionals

13. **Can we talk about your future expectations regarding living with long term indwelling urinary catheter and your quality of life with an IUC?**
    - What are your thoughts about using the catheter in the future?
    - What aspects of your catheter and/or drainage system would you like to see changed in the future?
    - What help do you think you will need with your catheter in the future?

End

Thank you for taking your time to share your experiences with me.
